# Opposing Roles for the Related ETS-Family Transcription Factors Spi-B and Spi-C in Regulating B Cell Differentiation and Function

**DOI:** 10.3389/fimmu.2020.00841

**Published:** 2020-05-08

**Authors:** Anne-Sophie Laramée, Hannah Raczkowski, Peng Shao, Carolina Batista, Devanshi Shukla, Li Xu, S. M. Mansour Haeryfar, Yodit Tesfagiorgis, Steven Kerfoot, Rodney DeKoter

**Affiliations:** ^1^Department of Microbiology and Immunology, Center for Human Immunology, Schulich School of Medicine and Dentistry, Western University, London, ON, Canada; ^2^Division of Genetics and Development, Children’s Health Research Institute, Lawson Research Institute, London, ON, Canada; ^3^Division of Clinical Immunology and Allergy, Department of Medicine, Western University, London, ON, Canada

**Keywords:** Spi-B, Spi-C, plasma cell, antibody, Bach2

## Abstract

Generation of specific antibodies during an immune response to infection or vaccination depends on the ability to rapidly and accurately select clones of antibody-secreting B lymphocytes for expansion. Antigen-specific B cell clones undergo the cell fate decision to differentiate into antibody-secreting plasma cells, memory B cells, or germinal center B cells. The E26-transformation-specific (ETS) transcription factors Spi-B and Spi-C are important regulators of B cell development and function. Spi-B is expressed throughout B cell development and is downregulated upon plasma cell differentiation. Spi-C is highly related to Spi-B and has similar DNA-binding specificity. Heterozygosity for *Spic* rescues B cell development and B cell proliferation defects observed in Spi-B knockout mice. In this study, we show that heterozygosity for *Spic* rescued defective IgG1 secondary antibody responses in *Spib*^–/–^ mice. Plasma cell differentiation was accelerated in *Spib*^–/–^ B cells. Gene expression, ChIP-seq, and reporter gene analysis showed that Spi-B and Spi-C differentially regulated *Bach2*, encoding a key regulator of plasma cell and memory B cell differentiation. These results suggest that Spi-B and Spi-C oppose the function of one another to regulate B cell differentiation and function.

## Introduction

Naïve B cells possess the capacity to differentiate into heterogeneous cellular subsets that promote the resolution of both current and future infections. These subsets include Plasma Cells (PC), memory B cells, and germinal center (GC) B cells (1, 2). The formation of GCs by GC B cells is crucial for the generation of robust humoral responses to T cell-dependent antigens, as this specialized microenvironment allows B cells to undergo class-switch recombination and affinity maturation (3, 4). Within the dark zone of the GC, rapidly cycling B cells accumulate point mutations in the variable regions of BCR-encoding immunoglobulin genes (5). This stochastic process, known as somatic hypermutation, diversifies the range of antigenic affinities exhibited by GC B cells (6).

The molecular determinants responsible for regulating B cell fate decisions following GC entry have become the focus of intense scrutiny. The transcription factors Bcl-6 and Blimp-1 (encoded by *Bcl6* and *Prdm1*, respectively) exert opposing activities on B cell differentiation. Blimp-1 coordinates the silencing of B cell gene expression patterns by repressing *Pax5* (7, 8). In contrast, Bcl6 promotes the generation and maintenance of GCs by repressing *Prdm1* (9, 10). Interferon regulatory factors 4 (IRF4) and 8 (IRF8) govern the fate of activated B cells in a concentration-dependent manner (11). High intracellular abundance of IRF4 (paired with low levels of IRF8) promote the generation of plasmablasts and PCs, while high IRF8 and correspondingly low IRF4 expression promote the GC fate (11). Therefore, transcription factors regulate PC differentiation versus GC differentiation through networks involving mutually cross-antagonistic activity.

Spi-C (encoded by *Spic*), Spi-B (encoded by *Spib*) and PU.1 (encoded by *Spi1*) are related E26-transformation-specific (ETS) transcription factors that are critically important at multiple stages of B cell development (12, 13). PU.1 and Spi-B function as complementary transcriptional activators of genes involved in B cell development and function (14–16). PU.1 and Spi-B expression are required for secondary Ig responses in mice (17, 18). In contrast, down-regulation of PU.1 and Spi-B expression is required for PC differentiation (19, 20). Forced expression of Spi-B has been shown to inhibit PC differentiation (20). Spi-C is expressed in pre-B and mature B cells as well as in red pulp macrophages (21–23). Spi-C functions primarily as a transcriptional repressor, and may antagonize the activities of PU.1 and Spi-B (24–27). Deletion of one allele of *Spic* was found to partially rescue B cell development, and proliferation of cultured *Spib*^–/–^ B cells in response to LPS or anti-IgM (28). However, the role of Spi-C in regulating B cell function including antibody responses has not been investigated.

Bach1 and Bach2 are related basic region leucine zipper proteins that function as transcriptional repressors (29). Bach1 and Bach2 de-repress target genes upon interaction with heme (30). Bach1 is highly expressed in the myeloid lineage, while Bach2 is expressed in the B cell and T cell lineages (31). Bach2 is required for the GC and memory B cell fates (32, 33), while down-regulation of Bach2 is required for PC differentiation (34, 35). Bach1 and Bach2 transcriptionally repress the myeloid gene program in macrophages and lymphocytes (36, 37). Bach1 represses *Spic* transcription in myeloid cells (38). De-repression of *Spic* transcription by heme-induced Bach1 degradation is required for differentiation into red pulp macrophages (38). The Heme-Bach1-Spi-C pathway has emerged as an paradigm for how an external signal can instruct lineage cell fate decisions through a cell type specific transcription factor (21, 38).

In this study, we show that deletion of one allele of *Spic* rescued IgG1 secondary antibody responses in *Spib*^–/–^ mice. Differentiation of *Spib*^–/–^ B cells into plasmablasts was accelerated in culture. Gene expression and ChIP-seq analysis showed that Spi-B and Spi-C differentially regulated the target gene *Bach2* that is a key regulator of secondary antibody responses and PC differentiation. These results suggest that Spi-C is a negative regulator of Spi-B activity, and that both proteins are important regulators of B cell fate decisions.

## Materials and Methods

### Mice

*Spib*^–/–^ and *Spib*^–/–^*Spic* ^+ ⁣/−^ mice were maintained as previously described (28). C57BL/6 (WT) mice were purchased from Charles River Laboratories (Pointe-Claire, QC, Canada). All animals were housed under specific pathogen-free conditions at the West Valley facility (London, ON, Canada), and were monitored in accordance with an animal use protocol approved by the Western University Council on Animal Care and the Animal Care Committee. Genotyping was performed by PCR, as previously described (12, 21). All experiments performed in this study used 6–10 week old mice. Mice were immunized i.p. with 100 μg of 4-Hydroxy-3-nitrophenylacetyl hapten (NP)-conjugated ovalbumin or keyhole limpet hemocyanin (KLH) (conjugation ratios of 15:1 NP:OVA, 8:1 NP:KLH) (Biosearch Technologies, Novato, CA, United States) adjuvanted with 50% (vol/vol) of Imject^TM^ alum (ThermoFisher Scientific, Rochester, NY, United States). For experiments involving secondary responses, mice were re-immunized by i.p. injection on day 30 following the primary challenge with a boosting dose identical to that of the prime.

### B Cell Enrichment and Culture

Splenocytes were enriched for B cells by negative selection using the VarioMACS magnet, LD depletion columns, streptavidin microbeads (Miltenyi Biotec, Germany) and biotin-conjugated mouse anti-CD43 (S7). Enriched B cells were stained using the CellTrace Violet Cell Proliferation Kit (ThermoFisher) at a concentration of 1.5 μM. Enriched B cells or murine 38B9 cells were cultured in were cultured in complete RPMI-1640 containing 10% fetal bovine serum (Wisent, St. Bruno, QC, Canada), 5 × 10^–^^5^ M β-mercaptoenthanol (Sigma-Aldrich, St. Louis, MO, United States), 0.01 M HEPES (Sigma-Aldrich), and 1X penicillin/streptomycin/L-glutamine (Wisent). WEHI-279 cells were cultured in complete DMEM medium containing 4.5 g/L glucose (Wisent). Cells were maintained in 5% CO_2_ at 37°C. B cells were stimulated with LPS 0111:B4 (10 μg/ml, List Biological Laboratories, Campbell, CA, United States), 100 ng/ml CD40L (R&D Systems, Minneapolis, MI, United States), 10 ng/ml Interleukin-4 (R&D Systems), and/or 10 ng/ml Interleukin-5 (R&D Systems). Cultured plasmablasts were analyzed by flow cytometry on day 3, 4, or 5 of culture.

### Luciferase Assays

*Bach2* region of interest 1 (ROI 1) was PCR amplified from murine genomic DNA using Q5 high-fidelity DNA polymerase (New England Biolabs, Ipswich, MA, United States). PCR products were cloned using the StrataClone Blunt PCR cloning kit (Agilent Technologies, La Jolla, CA, United States). ROI 1 was ligated in the forward orientation into the *Kpn*I/*Sac*I sites of the luciferase reporter pGL3-promoter (Promega, Madison, WI, United States) and confirmed by sequencing. Mutation of the predicted ROI 1 ETS site (GGAA → GGCC) was performed using the Q5 site directed mutagenesis kit (New England Biolabs). pRL-TK (Renilla luciferase), pGL3-basic, pGL3-promoter, pGL3-promoter-ROI 1, and pGL3-promoter ROI 1 mutant vectors were transfected into 4 × 10^6^ WEHI-279 cells by electroporation using a Gene Pulser II with capacitance extender at 220V and 950 mF (Bio-Rad, Mississauga, ON, United States). Luciferase activity was measured 24 h after transfection using the Dual Luciferase Assay Kit (Promega). Luminescence was determined using a Cytation 5 plate reader (BioTek, Winooski, VT, United States).

### ELISpot Assays

Single-cell suspensions of splenocytes were red blood cell-depleted using ammonium-chloride-potassium (ACK) lysis, washed in MACS buffer and counted using the Moxi Z Mini Automated Cell Counter (Orflo, Ketchum, ID, United States). Splenocytes were serially diluted and incubated for 5 h at 37°C (5% CO_2_) in 10% FBS-containing RPMI 1640X. Alkaline phosphatase-conjugated goat anti-mouse IgM (Mabtech), IgG1, IgG2b, and IgG2c (Jackson Immunoresearch, Westgrove, PA, United States) were incubated overnight in corresponding wells. 5-bromo-4-chloro-3-indolyl-phosphate (BCIP, Sigma-Aldrich) in 3% low-melt agarose gel (NuSieve^TM^ GTG^TM^ Agarose, Lonza, Basel, Switzerland) was used to develop spots. Plates were imaged using the ImmunoSpot^®^ S6 Analyzer and counted using the ImmunoSpot^®^ software (Cellular Technology Limited, Cleveland, OH). Spot counts from triplicate wells in individual mice were averaged, then plotted against the corresponding dilution for each antibody. Data points were calculated from logarithmic functions, obtained by plotting mean values of spots from triplicate wells against corresponding dilutions for each mouse. Logarithmic regressions were performed in Excel, and curves of best fit were used to calculate adjusted frequencies of ASCs per 1 × 10^6^ cells.

### Flow Cytometry

Anti-Fc-γ receptor blocking was performed using purified anti-CD16/CD32 (Mouse BD Fc Block). Cell-surface staining was performed using the following Abs, purchased from eBioscience (San Diego, CA, United States), BD Bioscience (Franklin Lakes, NJ, United States) or Biolegend (San Diego, CA, United States): Brilliant Violet 421 and allophycocyanin (APC)-conjugated anti-CD45R/B220 (clone Ra3-6B2); biotin, phycoerythrin (PE), or brilliant violet 421 (BV421)-conjugated anti-CD138 (clone 281-2); PE, fluorescein (FITC), or Alexa Fluor 488-conjugated CD19 (6D5); PE-conjugated anti-CD38 (clone 90); PE-Cy5-conjugated anti-CD4 (clone RM4-5); PE-Cy7 anti-CD95 (clone Jo2); and PE-conjugated streptavidin. Live/dead discrimination was performed using fixable viability dye eFluor 506 (eBioScience). Analysis was performed using BD FACSCanto or LSR II instruments (BD Immunocytometry Systems, San Jose, CA, United States). Cell sorting was performed using a FACSAria III instrument (BD Immunocytometry Systems) at the London, Ontario Regional Flow Cytometry Facility. Analysis was performed using FlowJo 10.4 (TreeStar, Ashland, OR, United States).

### Reverse Transcription Quantitative Polymerase Chain Reaction (RT-qPCR)

Splenic B cells were enriched from WT, *Spib*^–/–^ and *Spib*^–/–^*Spic* ^+ ⁣/−^ mice and cultured in complete RPMI supplemented with IL-4, IL-5, and CD40L for 4 days. Cultured B cells were enriched into CD138^+^ or CD138^–^ fractions using biotinylated anti-CD138 (281-2) and the Miltenyi system (Miltenyi Biotec, Germany). Total RNA was extracted using TRIzol (ThermoFisher) or the RNeasy Mini Prep Kit (Qiagen, Hilden, Germany). Following cDNA synthesis (iScript cDNA Synthesis Kit, Bio-Rad), RT-qPCR analysis was conducted using the SensiFAST SYBR No-ROX Kit (Bioline, Singapore) on the QuantStudio 5 Real-Time PCR System (ThermoFisher). Analyses were conducted in duplicates, with relative expression of target genes normalized to *Tata-binding protein (Tbp)*, and calculated as fold change using the comparative threshold cycle [2^(^^–^^ΔΔ^^*C*^_*T*_^)^] method (39). The selection of *Tbp* as a reference gene was carried out on the basis of its relative stability and high expression, by re-analysis of previously published RNA-seq data (GEO accession code: GSE60927) (40), in which the variance in log_2_FPKM values from sorted FO B cells, GC B cells, plasmablast and PC subsets was compared. Amplification efficiencies were calculated for each primer pair (Supplementary Table S1) using calibration curves generated by triplicate doubling dilutions of total splenocyte cDNA. Primer pairs with efficiencies ranging from 90 to 110% were used in the study.

### Production of Retrovirus and Primary B Cell Transduction

MIG-3XFLAG-SpiB and MIG-3XFLAG-SpiC retroviral vectors (15) were packaged by transient transfection of Platinum-E (Plat-E) retroviral packaging cells using polyethyleminine (PEIpro, PolyPlus, Illkirch, France) (41). Plat-E supernatant containing viral particles was harvested after 48 h, and transfection efficiency was analyzed by flow cytometry. Primary B cells were stimulated in CD40L+IL-4+IL-5 (R&D Systems) overnight. Transduction of stimulated, enriched B cells was performed by centrifugal infection at 3000 × *g* for 2 h at 32°C. Following transduction, primary B cells were cultured for 3 days in complete RPMI (Wisent) containing CD40L+IL-4+IL-5 (R&D Systems), as described above.

### Chromatin Immunoprecipitation

Chromatin was prepared from pellets of 1 × 10^6^ transduced, cultured B cells as described in (12). Cross-linking was performed using 1% formaldehyde (Millipore-Sigma, Darmstadt, Germany) and halted using glycine. Pellets were flash-frozen in liquid nitrogen prior to sonication. Thawed pellets were lysed in lysis buffer supplemented with Halt Protease Inhibitor (ThermoFisher Scientific, Rochester, NY, United States), and sonicated for 25 cycles using the Bioruptor UCD-300 (Diagenode, Sparta, NJ, United States). Immunoprecipitation of FLAG-bound chromatin was performed using anti-FLAG M2 magnetic beads (MilliporeSigma, Darmstadt, Germany). Eluted DNA was purified with QIAquick PCR Purification Kit (Qiagen, Hilden, Germany). qPCR on purified DNA was performed as described above, using primers shown in Supplementary Table S1. Threshold cycle values were used to calculate enrichment, represented as percent input. ROIs were identified by analysis of published ChIP-seq data (GEO accession code: GSE58128) (14). ChIP-seq was performed as described in Solomon et al. (14). Quality control for chromatin enriched by anti-FLAG antibody was performed by qPCR analysis for association with the IgH intronic enhancer. Sequencing was performed by Genome Quebec on two independent replicates of anti-FLAG ChIP chromatin as well as on input chromatin DNA.

### Bioinformatic and Statistical Analysis

ChIP-seq analysis was performed using the Galaxy Suite of bioinformatic tools (42). Bowtie2 was used to merge the two experimental samples and align reads to mouse genome Mm9 (43). Peaks were called using MACS (44) with the input as control, using a tag size of 70, a band width of 300, and a *p*-value cutoff of 1e^–^^5^. Peak-to-gene association was called using Cistrome, with a 15,000 bp cutoff (45). Gene Ontology analysis was performed using DAVID (46). Motif analysis was performed using MEME Suite 4.11.0 (47). The sequence of the *Bach2* locus was analyzed for multi-species conservation analysis (PhastCons46wayPlacental) using ORCAtk (Version 1.0.0), with the following settings: minimum conservation 70%, minimum conserved region 20. ChIP-seq data is available from the Gene Expression Omnibus accession GSE115593. Statistical analyses were performed using Prism 8.2 (GraphPad, San Diego, CA, United States) using specific tests described in figure legends.

## Results

### Heterozygosity for Spi-C Rescues Spib^–/–^ Defect in Secondary T Cell-Dependent B Cell Responses

**FIGURE 1:**
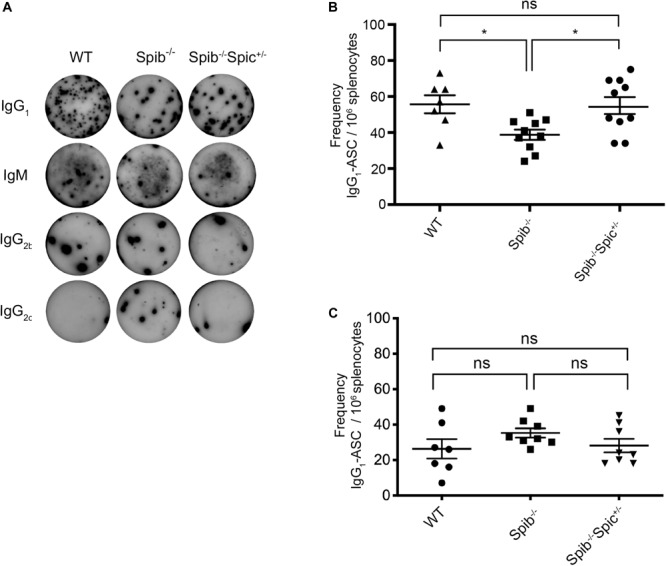
Differential regulation of secondary antibody responses by Spi-B and Spi-C. **(A)** Representative ELISpot wells for detection of IgG_1_, IgM, IgG2b, and IgG2c. **(B)** IgG_1_-ASC frequencies at day 37. Mice were immunized in 3 experiments of 3–4 mice of each genotype. Data are shown for *n* = 10 individual *Spib*^–/–^ and *Spib*^–/–^*Spic* ^+ ⁣/−^ mice, and *n* = 7 individual WT mice. **(C)** IgG1-ASC frequencies at day 7. Mice were immunized in 3 experiments of 2–3 mice of each genotype. Data are shown for *n* = 8 individual *Spib*^–/–^ and *Spib*^–/–^*Spic* ^+ ⁣/−^ mice, and *n* = 7 individual WT mice. Data are shown as mean ± SEM. Statistics were determined by one-way ANOVA with Tukey’s multiple comparisons test, *p < 0.05.

Mice homozygous for a null allele of *Spib* on a BALB/c background exhibit reduced titers of anti-nitrophenyl (NP) antibodies following secondary challenge with nitrophenyl (NP) conjugated to keyhole limpet hemocyanin (KLH) (18). We previously reported that heterozygosity for *Spic* rescued B cell development and proliferation of *Spib*^–/–^ B cells in response to TLR stimulation. We sought to determine whether heterozygosity for *Spic* could rescue the impairment in antibody-secreting cell (ASC) frequencies in *Spib*^–/–^ mice on a C57BL/6 background immunized with NP conjugated to ovalbumin (OVA). *Spib*^–/–^ *Spic* ^+ ⁣/−^ mice or *Spic* ^+ ⁣/−^ mice could not be included due to the high frequency of embryonic lethality in these mice (28). WT, *Spib*^–/–^, and *Spib*^–/–^*Spic* ^+ ⁣/−^ mice were immunized intraperitoneally with a priming dose of alum-precipitated NP-OVA. An identical challenge was administered at day 30 post-immunization, and splenic frequencies of ASCs were assessed by Enzyme-Linked ImmunoSpot (ELISpot) at day 37 post-immunization. For *Spib*^–/–^ and *Spib*^–/–^*Spic* ^+ ⁣/−^ immunized littermates, we observed IgG_1_ responses of greater magnitude than IgM, and very low frequencies of B cells of other isotypes, which was expected based on the known dominance of IgG1 utilizing VH186.2-DFL16.1-JH2 and l1 in anti-NP responses (48) (Figure 1A and Supplementary Figures S1A–C). There was a significant reduction in frequencies of NP-reactive, IgG_1_-forming or IgM-forming ASCs in immunized *Spib*^–/–^ mice relative to WT controls (Figure 1B and Supplementary Figure S1A). Heterozygosity for *Spic* in *Spib*^–/–^*Spic* ^+ ⁣/−^ mice increased IgG_1_ ASC frequencies to WT levels (Figure 1B), but did not increase IgM ASC frequencies.

Since the day 37 response represents a combination of the 7-day primary response and the boosted 30-day response, we also measured the primary response of WT, *Spib*^–/–^, and *Spib*^–/–^*Spic* ^+ ⁣/−^ mice using immunization with NP-OVA in alum followed by ELISPOT analysis at day 7 post-immunization. At this time point there were no significant differences between NP-reactive ASCs in responding *Spib*^–/–^ and *Spib*^–/–^*Spic* ^+ ⁣/−^ animals for IgG_1_ (Figure 1C), IgM, IgG_2__*b*_, and IgG_2__*c*_ isotypes (Supplementary Figures S1D–F). We conclude that the significant difference observed in day 37 IgG1 or IgM responses represents a difference in the recall response to NP-OVA immunization, rather than in the primary response. Reduced secondary responses in *Spib*^–/–^ mice, that were rescued in *Spib*^–/–^*Spic* ^+ ⁣/−^ mice, suggest a role for Spi-B and Spi-C in promoting either GC B cell differentiation or memory B cell differentiation.

### Accelerated Differentiation of Spib^–/–^ B Cells Into Plasmablasts in Culture

**FIGURE 2:**
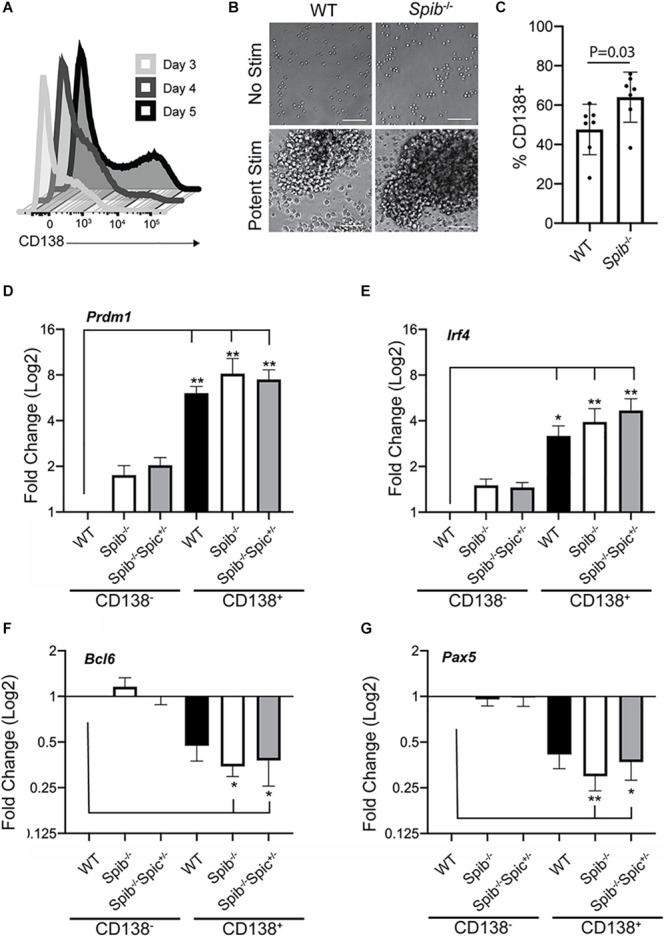
Plasmablast differentiation in cultures containing CD40L, IL-4, and IL-5. **(A)** Frequencies of CD138^+^ plasma cells following stimulation of enriched WT B cells for 3, 4, or 5 days with CD40L+IL-4+IL-5. One representative experiment is shown. **(B)** Photomicrographs of cell cultures, original magnification 20X. Scale bar indicates 50 μm. One representative experiment is shown. **(C)** Frequencies of CD138^+^ plasma cells following 5 days of culture. Means are shown ± SEM (*n* = 7 independent experiments with individual mice, unpaired *t*-test). **(D–G)** RT-qPCR analysis was performed for the indicated genes using total RNA prepared from CD138-enriched and -depleted samples, following 4 days of culture. Relative gene expression was normalized to *Tbp.* Fold change in expression was determined relative to WT CD138^-^ cells, and is shown as mean ± SEM (*n* = 7 independent experiments with individual mice of each genotype, Kruskal-Wallis and Dunn’s multiple comparisons test), *p < 0.05; **p < 0.01.

PU.1 and Spi-B expression are down-regulated upon PC differentiation (40). Combined deficiency in PU.1 and Spi-B increases PC differentiation (19, 49). Immature PCs (plasmablasts) can be generated in culture using stimulation with CD40L, Interleukin-4 (IL-4), and Interleukin-5 (IL-5) (50). Culture of splenic B cells, enriched to ∼97% using magnetic beads, with CD40L + IL-4 + IL-5 promoted differentiation into CD138^+^ plasmablasts with increasing frequency in 3–5 days (Figure 2A). When WT or *Spib*^–/–^ splenic B cells were enriched and cultured with CD40L+IL-4+IL-5 for 5 days, frequencies of CD138-expressing cells in *Spib*^–/–^ cultures were significantly elevated compared to WT samples (Figures 2B,C). To further validate this culture system, enriched splenic B cells from WT, *Spib*^–/–^ and *Spib*^–/–^*Spic* ^+ ⁣/−^ mice were cultured with CD40L+IL-4+IL-5 for 4 days in order to generate equivalent numbers of CD138^+^ and CD138^–^ cells (Figure 2A). Cultured cells were enriched for CD138^+^ and CD138^–^ cells using magnetic beads. Total RNA was isolated and used to determine mRNA transcript levels of *Prdm1*, *Irf4*, *Bcl6*, and *Pax5* using reverse transcription quantitative PCR (RT-qPCR). Transcript levels were normalized to TATA-binding protein (*Tbp*). We found mRNA transcript levels of *Prdm1* (encoding Blimp-1) and *Irf4* (encoding Interferon-Response Factor-4) to be increased in CD138^+^ enriched cells relative to that of the WT CD138-depleted fraction (Figures 2D,E). Differentiation of B cells into CD138^+^ cells was accompanied by downregulation of *Bcl6*, *Pax-5* mRNA compared to CD138^–^ cells (Figures 2F,G). These patterns of gene expression confirmed differentiation of B cells into plasmablasts under these culture conditions (51).

**FIGURE 3:**
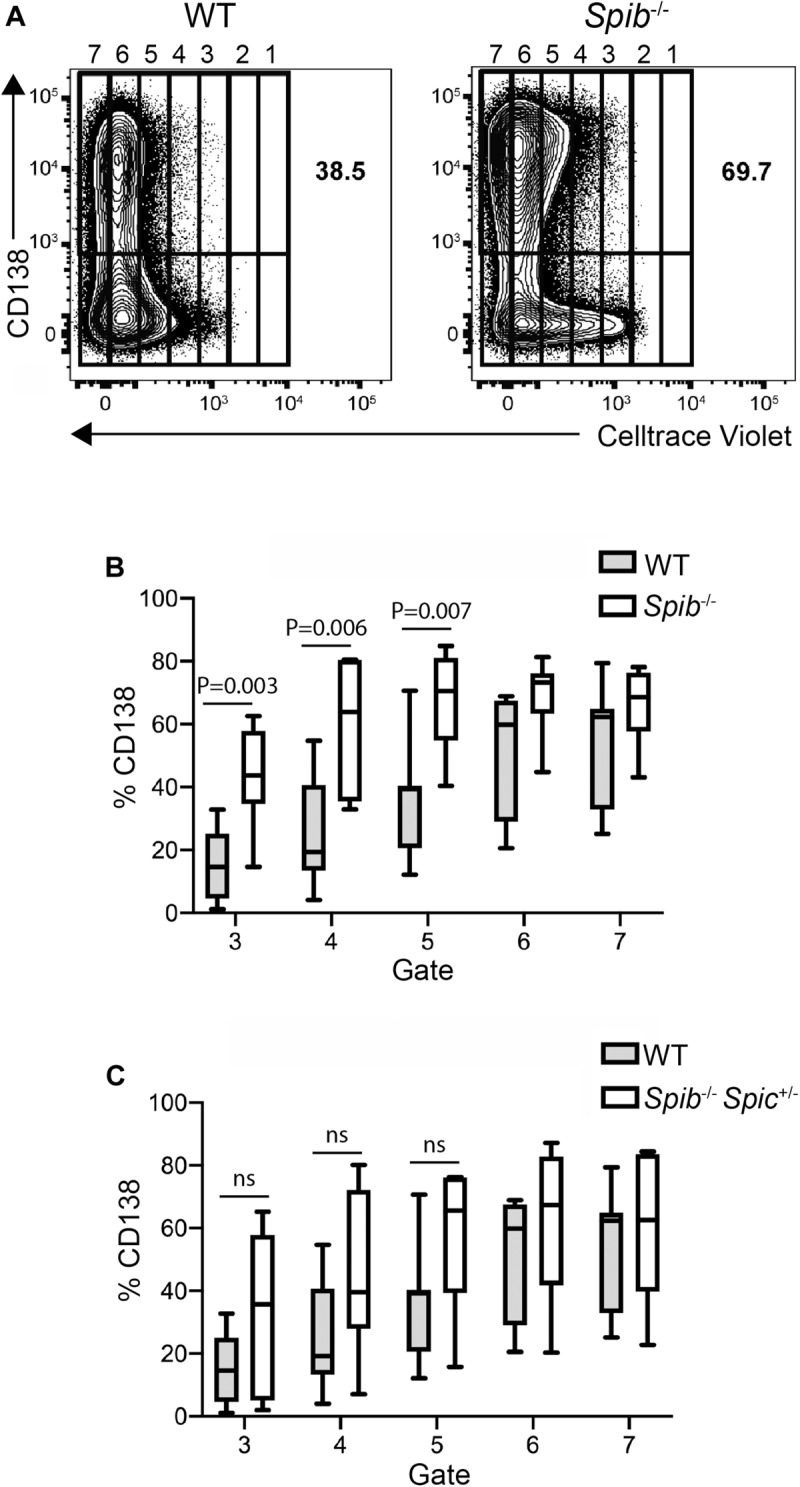
Accelerated plasmablast generation from *Spib*^–/–^ B cells. **(A)** Increase in plasma cell frequency in cultured *Spib*^–/–^ cells. Frequencies of CD138^+^ plasma cells were quantified in WT and *Spib*^–/–^ enriched B cells, following 5 days of culture. Cells were assigned to arbitrary gates to track cell division. The rightmost gate denotes undivided cells. One representative experiment is shown. **(B,C)** Frequencies of CD138^+^ plasma cells were quantified in gates 3–7 for WT, *Spib*^–/–^ and *Spib*^–/–^Spic ^+ ⁣/−^ enriched B cells, following 5 days of culture. Data are shown as box-and-whisker plots in which the box represents the first and third quartile and the line represents the median (*n* = 7 independent experiments with individual mice of each genotype, one-way ANOVA and Holm–Sidak test).

To explore the kinetics of differentiation in successive rounds of cell division, WT, *Spib*^–/–^, or *Spib*^–/–^*Spic* ^+ ⁣/−^ splenic B cells were loaded with CellTrace Violet and cultured with CD40L+IL-4+IL-5 for 5 days. Cells were divided into gates based on Celltrace violet staining, with cells in gate 1 indicating no divisions and cells in gate 7 having divided the greatest number of times (Figure 3A). This analysis revealed a marked increase in the proportion of *Spib*^–/–^ CD138^+^ plasmablasts in the 3rd, 4th, and 5th gate, compared to WT cultures (Figures 3A,B). In contrast, *Spib*^–/–^*Spic* ^+ ⁣/−^ B cells showed no significant differences from WT in any gate, suggesting a phenotype intermediate between WT and *Spib*^–/–^ (Figure 3C). These results suggest that Spi-B, and to a lesser extent Spi-C, play roles in regulating plasmablast differentiation in response to CD40L+IL-4+IL-5.

### Evidence for Genetic Interaction of Spi-B, Spi-C, and Bach2

**FIGURE 4:**
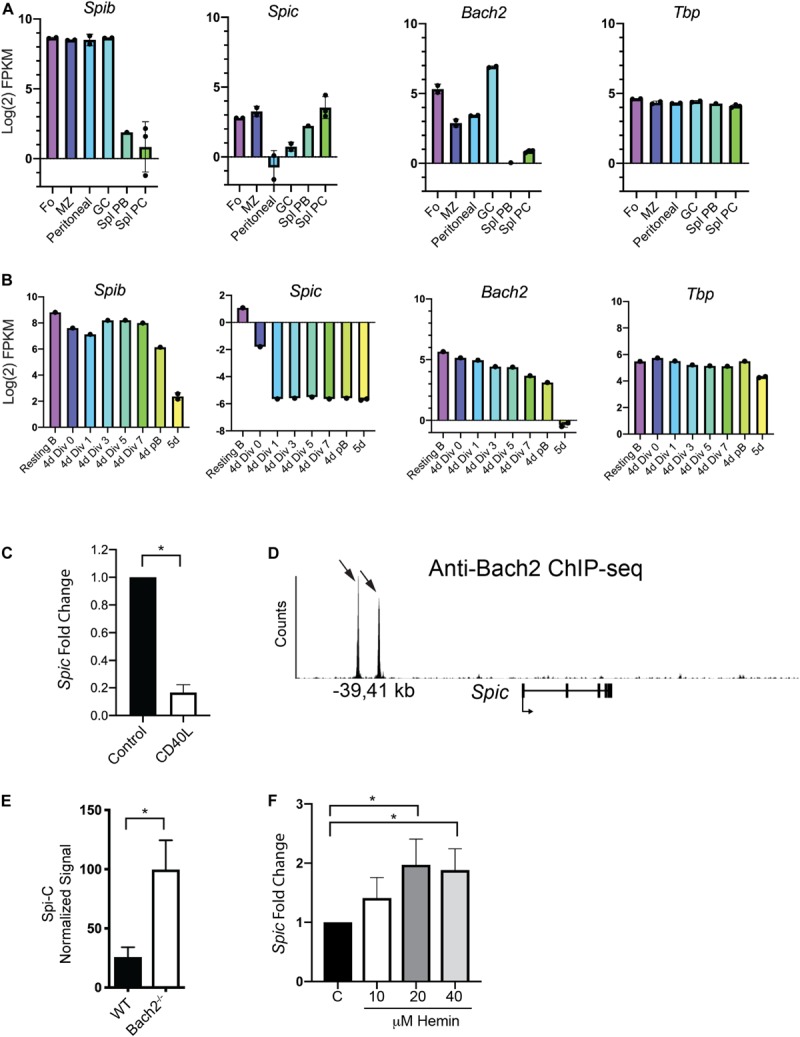
Evidence for repression of *Spic* by Bach2. **(A)** Analysis of gene expression in sorted mouse B cell populations. RNA-seq data from (40) was re-analyzed for enriched B cell populations including Follicular (Fo), Marginal Zone (MZ), Peritoneal, Germinal Center (GC), Splenic Plasmablast (Spl PB), and Splenic Plasma Cell (Spl PC). *Y*-axis shows gene expression as Log_2_ Fragments per Kilobase of transcript per Million mapped reads (FPKM). Dots indicate biological replicates. **(B)** Analysis of gene expression in cultured plasmablasts enriched based on cell division number. RNA-seq data from (40) was re-analyzed to compare gene expression in Resting B cells; B cells cultured for 4 days in CD40L+IL-4+IL-5 and sorted for 0, 1, 3, 5, or 7 cell divisions; CD138^+^ plasmablasts from 4 day cultures, and CD138^+^ plasmablasts from 5 day cultures (5 days). Dots indicate biological replicates. **(C)** Reduced *Spic* mRNA expression upon 24 h culture with CD40L. Control represents *Spic* mRNA expression in freshly enriched splenic B cells. Result is shown as mean ± SEM (*n* = 6 independent experiments with individual mice of each genotype, one sample *t* and Wilcoxon test) *p < 0.05. **(D)** Interaction of Bach2 with regulatory regions in the *Spic* locus. ChIP-seq data from (37) was re-analyzed to show interaction of Bach2 with a putative regulatory element located at –39 and –41 kb upstream of the *Spic* transcription start site. Black arrows indicate locations of Bach2 binding sites. **(E)** Increased *Spic* mRNA expression in anti-IgM-stimulated splenic B cells lacking Bach2. Agilent microarray data from (52) was re-analyzed; *y*-axis shows *Spic* normalized signal. Result is shown as mean ± SEM (*n* = 3 biological replicates, unpaired *t*-test), *p < 0.05. **(F)** Increased *Spic* mRNA transcript levels upon culture of B cells with hemin. Enriched splenic B cells were cultured with CD40L+IL-4+IL-5 and concentrations of hemin indicated on the *x*-axis. *Spic* mRNA transcript levels were determined using RT-qPCR of RNA prepared after 4 days of culture. Result is shown as mean ± SEM (*n* = 6 independent experiments with individual mice, Kruskal-Wallis and Dunn’s Multiple Comparisons test), *p < 0.05.

Bach1 and Bach2 are related basic region leucine zipper proteins that de-repress target genes upon interaction with heme (30). Bach1 and Bach2 transcriptionally repress target genes including *Spic* in common lymphoid progenitors (36, 37). In macrophages, heme-induced Bach1 degradation promotes differentiation into red pulp macrophages in a Spi-C-dependent manner (38). B cells express Bach2, that been shown to be a key regulator of the MBC versus PC fate decision (35). Whether Bach2 represses *Spic* in the B cell lineage to regulate B cell differentiation has not been investigated. We examined patterns of expression of *Spib*, *Spic*, and *Bach2*, using published RNA-seq data from enriched murine B cell populations including splenic PCs or cultured plasmablasts (40). *Spib* was highly expressed in all B cell subsets, and was downregulated upon PC differentiation (Figure 4A, left panel). *Spic* was expressed in Fo and MZ B cells, was expressed at low levels in peritoneal B1 and GC B cells, and was upregulated during PC differentiation with highest expression in PCs (Figure 4A, left panel). *Bach2* was expressed in all B cell subsets, with maximal expression in GC B cells, and was downregulated upon PC differentiation (Figure 4A, right panels). *Tbp* was stably expressed across all B cell subsets (Figure 4A, right panel). *Bach2* expression correlated with *Spib* expression (*r* = 0.89, *p* = 0.03 by Spearman’s test) but did not correlate with *Spic* expression.

Next, we examined published RNA-seq data from splenic B cells stimulated with CD40L+IL-4+IL-5, then sorted based on cell division (40). *Spib* and *Bach2* were downregulated over the course of PC differentiation (Figure 4B, first and third panel). In contrast to its pattern of expression in freshly enriched cells, *Spic* was sharply downregulated in culture with CD40L+IL-4+IL-5 (Figure 4B, second panel), while *Tbp* was stably expressed across all cell divisions (Figure 4B, fourth panel). *Spic* therefore showed a different pattern of expression in culture compared to freshly isolated populations, as it was expressed at high levels in splenic plasmablasts and PCs (Figure 4A) but was nearly undetectable in cultured plasmablasts (Figure 4B). To determine if *Spic* mRNA levels are regulated by CD40L, RNA was prepared from freshly enriched splenic B cells, or enriched B cells cultured 24 h with CD40L. RT-qPCR analysis showed that *Spic* was downregulated upon culture with CD40L (Figure 4C). This result suggests that CD40 signaling may explain differences in *Spic* expression in mice compared to cell culture, and may explain the low impact of *Spic* heterozygosity on plasma cell differentiation in cultures containing CD40L+IL-4+IL-5.

Next, we investigated whether Bach2 can repress *Spic* in B cells. Examination of Bach2 ChIP-seq analysis (36) demonstrated that Bach2 interacts directly with two sites located within upstream regulatory elements within the *Spic* locus (Figure 4D). To determine if *Spic* is repressed by Bach2 in B cells, we examined published microarray data from WT and *Bach2*^–/–^ B cells activated by anti-IgM stimulation (52). This analysis showed that *Spic* is up-regulated by approximately 4-fold in *Bach2*^–/–^ B cells compared to WT B cells (Figure 4E). Finally, Bach2 is a heme-binding protein, and its repressor activity is decreased by interaction with heme (30). Culture of splenic B cells with hemin in addition to CD40L+IL-4+IL-5 increased *Spic* mRNA transcript levels (Figure 4F). These results suggest that Bach2 is a transcriptional repressor of *Spic* in B cells.

### Chromatin Immunoprecipitation-Sequencing Analysis of Spi-C Binding in B Cells

**FIGURE 5:**
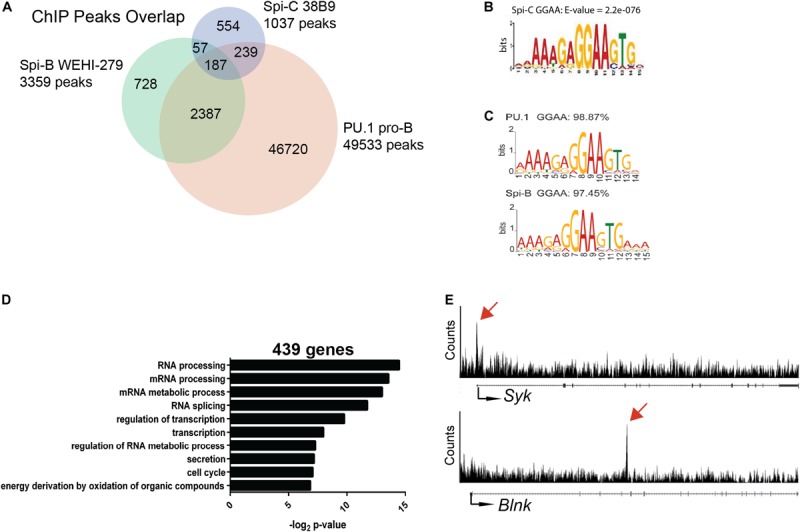
ChIP-seq analysis of Spi-C interaction in 38B9 cells. **(A)** Venn diagram showing overlap in binding sites. Genomic regions from ChIP analysis (peaks) were compared for Spi-C in 38B9 cells (blue), PU.1 in pro-B cells (red) and Spi-B in WEHI-279 B lymphoma cells (green). Numbers indicate the number of unique or overlapping peaks. **(B)** DNA interaction motifs recovered from Spi-C ChIP-seq analysis. **(C)** DNA interaction motifs recovered from PU.1 (top) and Spi-B (bottom) ChIP-seq analysis. **(D)** Biological pathways were identified from 439 genes interacting with Spi-C using DAVID analysis. **(E)** Representative Spi-C peaks in 38B9 cells for genes encoding *Syk* (top panel) and B cell linker protein (*Blnk*, lower panel). Red arrows indicate location of Spi-C peaks.

Genome-wide analysis of Spi-C interaction in the genome of B cells has not previously been reported because chromatin immunoprecipitation (ChIP)-grade antibodies for this transcription factor are not available. To determine Spi-C binding sites in the genome of B cells, we generated Abelson-transformed 38B9 pre-B cell lines infected with a retroviral vector encoding 3XFLAG-tagged murine Spi-C (12). Anti-FLAG ChIP-seq was performed, and reads were mapped to mouse genome Mm9 to identify binding sites. There were 1037 Spi-C binding sites identified using a *e* value cutoff of ≤10^–^^5^ (Figure 5A). These sites were then intersected with those previously identified for PU.1 in pro-B cells (13) and Spi-B in WEHI-279 cells (14). 554 peaks were unique to Spi-C only, 239 peaks were shared with PU.1; 57 peaks were shared with Spi-B; and 187 peaks were shared with both PU.1 and Spi-B (Figure 5A). For Spi-C peaks shared with either PU.1 or Spi-B, the most frequently occurring motif was a purine-rich sequence closely resembling the motif previously published for these transcription factors (Figures 5B,C) (14).

To identify genes regulated by Spi-C, Cistrome Beta Minus (53) was used to identify genes with transcription start sites located within 15 kb of Spi-C binding peaks. 439 genes were identified to be associated with Spi-C interaction. Among these, 56 genes were unique to Spi-C, while 383 were shared with either PU.1 or Spi-B. Gene ontology analysis was performed to identify biological pathways associated with genes uniquely occupied by Spi-C. The top three pathways included *RNA processing*, *mRNA processing*, and *mRNA metabolic process* (Figure 5D). Genes with Spi-C/Spi-B/PU.1 shared binding sites included *Syk* and *Blnk*, genes for which these transcription factors have been demonstrated to have opposing functions (15, 26) (Figure 5E, upper panels). These results suggested that half of Spi-C binding sites are coincident with PU.1 and/or Spi-B binding sites, but Spi-C also has unique binding sites and therefore may exert unique functions in B cells.

### Regulation of Bach2 by Spi-B and Spi-C

**FIGURE 6:**
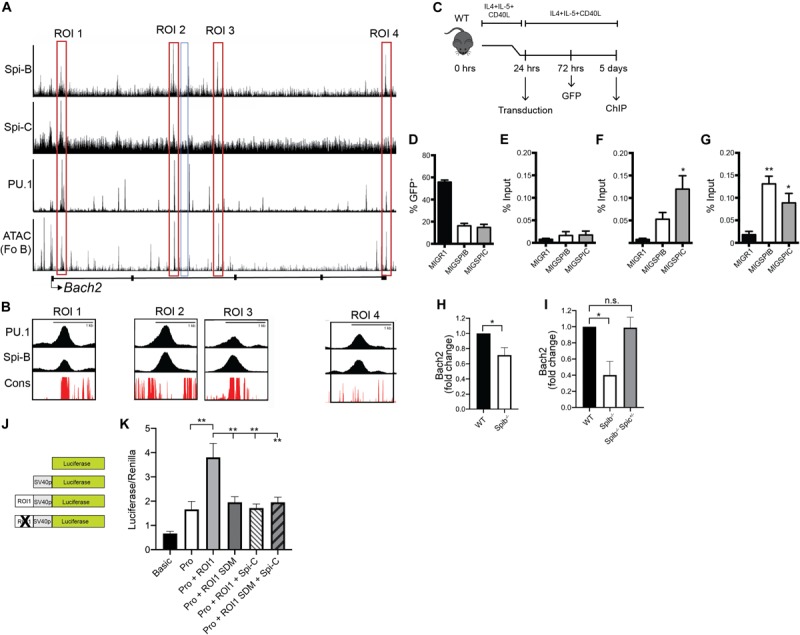
Regulation of *Bach2* by Spi-B and Spi-C. **(A)** UCSC genome browser tracks of ChIP-seq analysis of 3XFLAG-tagged Spi-B in WEHI-279 cells (top panel) and Spi-C in 38B9 cells (second panel). Also shown is ChIP-seq analysis of 3X-FLAG-tagged PU.1 binding in pro-B cells (third panel) and IMMGEN ATAC-seq analysis performed in enriched follicular B cells (Fo B) (Fourth panel). *Bach2* gene structure is shown below, with black boxes denoting exons and lines representing introns. Red boxes indicate regions-of-interest (ROIs 1-4), while the blue box denotes the negative control region (NCR). **(B)** Multiple placental species conservation analysis (in red) visualized as superimposed UCSC tracks of ChIP-seq (ROI 1-4, in black) within the *Bach2* locus for PU.1 and Spi-B. **(C)** Schematic depicting the workflow for primary B cell transduction, followed by chromatin immunoprecipitation (ChIP). **(D)** Frequency of GFP^+^ cells, 48 h following transduction of WT enriched B cells with the indicated retroviral vectors. Bars represent the mean transduction efficiency ± SEM. **(E–G)** Enrichment of FLAG-tagged Spi-B and Spi-C in cultured WT B cells at the NCR **(E)**, ROI 1 **(F)**, and ROI 3 **(G)**. Results are shown as percentage of input DNA, with bars representing mean% input ± SEM (*n* = 3 independent experiments using cells from individual mice, one-way ANOVA with Tukey’s multiple comparisons test), *p < 0.05; **p < 0.01. **(H)** Reduced *Bach2* mRNA transcript levels in *Spib*^–/–^ B cells. RT-qPCR analysis was performed on RNA prepared from splenic B cells enriched from WT and *Spib*^–/–^ mice (*n* = 5 independent experiments using individual mice of each genotype, one sample t and Wilcoxon test), *p < 0.05. **(I)** Reduced *Bach2* mRNA transcript levels in *Spib*^–/–^ GC B cells. RT-qPCR analysis was performed on RNA prepared from splenic GC B cells enriched from WT, *Spib*^–/–^, and *Spib*^–/–^ *Spic* ^+ ⁣/−^ mice by cell sorting, 10 days after immunization with NP-KLH. Result is shown for representative experiment with triplicate technical replicates, Kruskal-Wallis with Dunn’s multiple comparisons test, *p < 0.05. **(J)** Schematic of luciferase reporters pGL3-basic (first panel), pGL3-promoter (second panel), pGL3-promoter-ROI 1 (third panel), and pGL3-promoter-RO1 1 mutant (fourth panel). **(K)** *Bach2* ROI 1 shows enhancer activity in WEHI-279 cells that is reduced by mutation of the ETS binding site (*n* = 4 independent experiments using triplicate technical replicates, mixed-effects ANOVA with Tukey’s multiple comparisons test), **p < 0.01.

Next, our goal was to determine if Spi-B and Spi-C interact with regions in the *Bach2* locus. Four regions of interest (ROI 1-4) were identified in the *Bach2* locus based on regions of open chromatin marked by IMMGEN ATAC-seq analysis in enriched follicular B cells (54) (Figure 6A). ROI 1-4 were found to interact to differing extents with PU.1, Spi-B, and Spi-C based on the ChIP-seq analysis described above (Figure 6A). Multiple species conservation analysis (PhastCons46wayPlacental) of ROIs revealed a high degree of conservation in ROIs 1 and 3, found in *Bach2* introns 1 and 2, respectively (Figure 6B). To determine if Spi-B and Spi-C could interact with ROI 1 and 3 in primary B cells, splenic B cells from WT mice were enriched and stimulated overnight with CD40L+IL-4+IL-5, then transduced with MIGR1 (control), MIG-3XFLAG-SpiB, or MIG-3XFLAG-SpiC retroviral vectors (Figure 6C). Mean transduction efficiency, as determined by percentage of GFP^+^ cells, was 56% in MIGR1-transduced cells, 16% in MIG-3XFLAG-Spi-B-infected cells, and 15% in MIG-3XFLAG-Spi-C-infected cells (Figure 6D). Chromatin from transduced and stimulated B cells was immunoprecipitated using anti-FLAG antibodies and used as input for qPCR targeting ROIs 1 and 3, and on a negative control region (NCR) selected from intron 2 of *Bach2.* QPCR Analysis revealed significant enrichment of Spi-C at ROI 1 (Figure 6F) and ROI 3 (Figure 6G), relative to the NCR (Figure 5E) and also compared to that of MIGR1-transduced cells (Figures 6E–G). Spi-B interaction was likewise observed at ROI 1 and 3, compared to basal MIGR1 and NCR enrichment (Figures 6E–G). Therefore Spi-B and Spi-C can interact with ROI 1 and ROI 3 in B cells.

To determine if Spi-B is an activator of *Bach2*, we measured *Bach2* mRNA levels in freshly isolated *Spib*^–/–^ splenic B cells. We observed reduced *Bach2* mRNA levels in B cells enriched from the spleen of *Spib*^–/–^ mice (Figure 6H). Based on the observation that both Spi-B and Bach2 are expressed at high levels in GC B cells (Figure 4A), we performed the following experiment. WT, *Spib*^–/–^, and *Spib*^–/–^ *Spic* ^+ ⁣/−^ mice were immunized with alum-precipitated NP-KLH. Ten days after immunization, a time point reported to have high frequencies of GC B cells following immunization (17), CD38^–^ CD95^+^ CD19^+^ CD138^–^ splenic GC B cells (55) were enriched from the spleen of immunized mice by cell sorting. RT-qPCR analysis showed that *Bach2* mRNA transcripts were reduced in *Spib*^–/–^ GC B cells relative to WT GC B cells (Figure 6I). In contrast, *Bach2* mRNA transcripts were not reduced in *Spib*^–/–^ *Spic* ^+ ⁣/−^ GC B cells relative to WT GC B cells (Figure 6I). These data suggest that Spi-B and Spi-C regulate *Bach2* expression in naïve and GC B cells.

Bach2 ROI 1 was cloned and tested for enhancer activity in combination with a minimal SV40 promoter in WEHI-279 B lymphoma cells that express high levels of Spi-B, but do not express Spi-C (14) (Figure 6J). ROI 1 increased activity of the luciferase reporter (Figure 6K). Mutation of the ETS site (GGAA → GGCC) reduced activity of ROI 1 (Figure 6K). Co-transfection with a Spi-C expression vector repressed activity of the wild-type ROI 1 reporter, but did not repress activity of the mutant ROI 1 reporter plasmid (Figure 6K). Taken together, these data suggest that Spi-B is a transcriptional activator of *Bach2* through a binding site in ROI 1, while Spi-C can function as a transcriptional repressor at this site.

## Discussion

The goal of this study was to determine the roles of Spi-B and Spi-C in regulation of B cell antibody responses. Our results demonstrate that Spi-B and Spi-C play opposing roles in secondary antibody responses and plasmablast differentiation. *Spic* heterozygosity rescued frequencies of IgG1 antibody-secreting cells following secondary antigenic challenge in *Spib*^–/–^ mice. Culture of enriched splenic B cells with CD40L+IL-4+IL-5 revealed that *Spib*^–/–^ B cells differentiated into CD138-expressing plasmablasts with accelerated kinetics relative to WT cells. Using ChIP-seq, ChIP, and luciferase assays, we showed that Spi-B and Spi-C interact with intronic regions of the *Bach2* locus to regulate transcription. Experiments indicated that Spi-B activated *Bach2*, Spi-C repressed *Bach2*, and Spi-C and Bach2 were mutually cross-antagonistic. Together, our results suggest that Spi-B and Spi-C are involved in shaping antibody-forming responses by influencing the differentiation of activated B cells.

Our results showed that secondary IgG1 antibody responses and GC B cell frequencies are reduced in *Spib*^–/–^ mice relative to WT mice, and are partially rescued by deletion of one *Spic* allele in *Spib*^–/–^*Spic* ^+ ⁣/−^ mice. In contrast, plasmablast differentiation in culture was accelerated in *Spib*^–/–^ B cells relative to WT B cells. These results suggest that Spi-B promotes GC and/or memory B cell differentiation, but inhibits plasmablast differentiation. These observations fit well with previous observations that Spi-B is required for sustaining germinal centers (17), while in contrast Spi-B is normally downregulated upon plasmablast differentiation (Figure 4A), and that ectopic expression of Spi-B impairs plasmablast differentiation (20). We expect that the explanation for reduced secondary antibody responses in *Spib*^–/–^ mice might be a combination of reduced GC and/or memory B cell generation in *Spib*^–/–^ mice, and a tendency for naïve *Spib*^–/–^ B cells to differentiate into plasmablasts instead of GC B cells. We speculate that Spi-B and Spi-C may regulate cell fate decisions of naïve or GC B cells to differentiate into GC, memory or plasmablast cells. However, answering this question will require further experiments performed at single cell resolution.

ChIP-seq studies for Spi-C have not been previously performed because commercially available antibodies recognizing Spi-C do not work in ChIP. Therefore, we performed anti-FLAG ChIP-seq of 3XFLAG-tagged Spi-C in 38B9 pre-B cells. Interestingly, we found that ectopic expression of Spi-C could not be sustained in WEHI-279 lymphoma cells because it induced high rates of apoptosis (data not shown). ChIP-seq analysis for Spi-C revealed a 5′-GGAA-3′ motif that was consistent with that described for PU.1 and Spi-B (14). Examples of genes bound by PU.1, Spi-B, and Spi-C were *Syk* encoding the SYK tyrosine kinase and *Blnk* encoding B cell linker protein, both of which are key mediators of BCR signaling (15, 26). Thus, our Spi-C ChIP analysis revealed unique and shared sites at which Spi-C can compete with PU.1 and/or Spi-B binding in the genome of B cells in order to function as a regulator of PU.1 and/or Spi-B function.

Spi-C function in B cell antibody-forming responses has not been previously studied. We found that *Spic* heterozygosity rescued frequencies of IgG1 antibody-secreting cells following secondary antigenic challenge in *Spib*^–/–^ mice. These results are consistent with previous observations suggesting that Spi-C functions to oppose activity of Spi-B (28). Recently, Spi-C was found to bind DNA cooperatively with BCLAF to displace PU.1 or Spi-B from binding sites in developing B cells (27). Interestingly, *Spic* mRNA levels were discovered to be expressed differently *in vivo* than in culture (Figures 4A,B). This difference was found to be due to downregulation of *Spic* mRNA expression by CD40L (Figures 4B,C). Conversely, we found that *Spic* mRNA levels were increased in stimulated B cells from *Bach2*^–/–^ mice (Figure 4E) or in cultures treated with heme (Figure 4G). These experiments suggest that Spi-C is dynamically regulated in response to external signals such as CD40L and heme. We speculate that Spi-C responsiveness to heme may play a role in generation of plasmablasts in response to hemolytic bacterial infections (56).

The results presented in this study suggest the following model. Spi-B may function as an activator of *Bach2* in B cells to promote efficient GC and memory B cell differentiation (33, 35, 57). Upon induction of the plasmablast differentiation program, *Spib* is downregulated, leading to downregulated *Bach2* and induction of the plasmablast differentiation program by de-repression of Blimp-1 and Spi-C (58). Increased Spi-C expression would help enforce the plasmablast differentiation program in part by repression of *Bach2*. CD40/CD40L engagement of activated B cells by T follicular helper cells might down-regulate *Spic* to promote the GC/memory B cell fate. This model provides a framework for further study.

In summary, B cell developmental decisions are governed by mutually cross-antagonistic transcription factor networks including IRF4 versus IRF8 (11) or Bach2 versus Blimp-1 (35, 58). Our results suggest that Spi-B versus Spi-C may represent nodes in an additional opposing transcription factor network governing B cell differentiation. Understanding the molecular circuitry that governs B cell fate decisions during immune responses may ultimately have important implications for the design of vaccination strategies.

## Data Availability Statement

The datasets generated for this study can be found in the ChIP-seq data is available from the Gene Expression Omnibus accession GSE115593.

## Ethics Statement

The animal study was reviewed and approved by the Western University Council on Animal Care Committee.

## Author Contributions

RD conceived the study. A-SL, HR, PS, CB, DS, LX, and YT performed the experiments and interpreted the data. SH, SK, and RD interpreted the data. A-SL and RD wrote the manuscript.

## Conflict of Interest

The authors declare that the research was conducted in the absence of any commercial or financial relationships that could be construed as a potential conflict of interest.

## References

[1] Nutt SL Hodgkin PD Tarlinton DM Corcoran LM. The generation of antibody-secreting plasma cells. *Nat Rev Immunol.* (2015) 15:160–71. 10.1038/nri3795 25698678

[2] De Silva NS Klein U. Dynamics of B cells in germinal centres. *Nat Rev Immunol.* (2015) 15:137–48. 10.1038/nri3804 25656706 PMC4399774

[3] Kerfoot SM Yaari G Patel JR Johnson KL Gonzalez DG Kleinstein SH Germinal center B cell and T follicular helper cell development initiates in the interfollicular zone. *Immunity.* (2011) 34:947–60. 10.1016/j.immuni.2011.03.024 21636295 PMC3280079

[4] Victora GD Nussenzweig MC. Germinal centers. *Annu Rev Immunol.* (2012) 30:429–57. 10.1146/annurev-immunol-020711-075032 22224772

[5] Allen CDC Ansel KM Low C Lesley R Tamamura H Fujii N Germinal center dark and light zone organization is mediated by CXCR4 and CXCR5. *Nat Immunol.* (2004) 5:943–52. 10.1038/ni1100 15300245

[6] Gitlin AD Shulman Z Nussenzweig MC. Clonal selection in the germinal centre by regulated proliferation and hypermutation. *Nature.* (2014) 509:637–40. 10.1038/nature13300 24805232 PMC4271732

[7] Shaffer AL Lin K-I Kuo TC Yu X Hurt EM Rosenwald A Blimp-1 Orchestrates Plasma Cell Differentiation by Extinguishing the Mature B Cell Gene Expression Program. *Immunity.* (2002) 17:51–62. 10.1016/S1074-7613(02)00335-7 12150891

[8] Lin K-I Angelin-Duclos C Kuo TC Calame K. Blimp-1-dependent repression of Pax-5 is required for differentiation of B cells to immunoglobulin M-secreting plasma cells. *Mol Cell Biol.* (2002) 22:4771–80. 10.1128/mcb.22.13.4771-4780.2002 12052884 PMC133916

[9] Crotty S Johnston RJ Schoenberger SP. Effectors and memories: Bcl-6 and Blimp-1 in T and B lymphocyte differentiation. *Nat Immunol.* (2010) 11:114–20. 10.1038/ni.1837 20084069 PMC2864556

[10] Ochiai K Muto A Tanaka H Takahashi S Igarashi K. Regulation of the plasma cell transcription factor Blimp-1 gene by Bach2 and Bcl6. *Int Immunol.* (2008) 20:453–60. 10.1093/intimm/dxn005 18256039

[11] Xu H Chaudhri VK Wu Z Biliouris K Dienger-Stambaugh K Rochman Y Regulation of bifurcating B cell trajectories by mutual antagonism between transcription factors IRF4 and IRF8. *Nat Immunol.* (2015) 16:1274–81. 10.1038/ni.3287 26437243

[12] DeKoter RP Geadah M Khoosal S Xu LS Thillainadesan G Torchia J Regulation of follicular B cell differentiation by the related E26 transformation-specific transcription factors PU.1, Spi-B, and Spi-C. *J Immunol.* (2010) 185:7374–84. 10.4049/jimmunol.1001413 21057087

[13] Batista CR Li SKH Xu LS Solomon LA DeKoter RP. PU.1 Regulates Ig light chain transcription and rearrangement in Pre-B cells during B cell development. *J Immunol.* (2017) 198:1565–74. 10.4049/jimmunol.1601709 28062693

[14] Solomon LA Li SKH Piskorz J Xu LS DeKoter RP. Genome-wide comparison of PU.1 and Spi-B binding sites in a mouse B lymphoma cell line. *BMC Genomics.* (2015) 16:76 10.1186/s12864-015-1303-0 PMC4334403 25765478

[15] Xu LS Sokalski KM Hotke K Christie DA Zarnett O Piskorz J Regulation of B cell linker protein transcription by PU.1 and Spi-B in murine B cell acute lymphoblastic leukemia. *J Immunol.* (2012) 189:3347–54. 10.4049/jimmunol.1201267 22956576

[16] Christie DA Xu LS Turkistany SA Solomon LA Li SK Yim E PU.1 opposes IL-7-dependent proliferation of developing B cells with involvement of the direct target gene bruton tyrosine kinase. *J Immunol.* (2015) 194:595–605. 10.4049/jimmunol.1401569 25505273

[17] Su GH Chen HM Muthusamy N Garrett-Sinha LA Baunoch D Tenen DG Defective B cell receptor-mediated responses in mice lacking the Ets protein. *Spi-B. EMBO J.* (1997) 16:7118–29. 10.1093/emboj/16.23.7118 9384589 PMC1170313

[18] Garrett-Sinha LA Su GH Rao S Kabak S Hao Z Clark MR PU.1 and Spi-B are required for normal B cell receptor-mediated signal transduction. *Immunity.* (1999) 10:399–408. 10.1016/s1074-7613(00)80040-0 10229183

[19] Willis SN Tellier J Liao Y Trezise S Light A O’Donnell K Environmental sensing by mature B cells is controlled by the transcription factors PU.1 and SpiB. *Nat Commun.* (2017) 8:1426 10.1038/s41467-017-01605-1 PMC5681560 29127283

[20] Schmidlin H Diehl SA Nagasawa M Scheeren FA Schotte R Uittenbogaart CH Spi-B inhibits human plasma cell differentiation by repressing BLIMP1 and XBP-1 expression. *Blood.* (2008) 112:1804–12. 10.1182/blood-2008-01-136440 18552212 PMC2518887

[21] Kohyama M Ise W Edelson BT Wilker PR Hildner K Mejia C Role for Spi-C in the development of red pulp macrophages and splenic iron homeostasis. *Nature.* (2009) 457:318–21. 10.1038/nature07472 19037245 PMC2756102

[22] Bemark M Martensson A Liberg D Leanderson T. Spi-C, a novel Ets protein that is temporally regulated during B lymphocyte development. *J Biol Chem.* (1999) 274:10259–67. 10.1074/jbc.274.15.10259 10187812

[23] Hashimoto S Nishizumi H Hayashi R Tsuboi A Nagawa F Takemori T Prf, a novel Ets family protein that binds to the PU.1 binding motif, is specifically expressed in restricted stages of B cell development. *Int Immunol.* (1999) 11:1423–9. 10.1093/intimm/11.9.1423 10464163

[24] Schweitzer BL Huang KJ Kamath MB Emelyanov AV Birshtein BK DeKoter RP. Spi-C has opposing effects to PU.1 on gene expression in progenitor B cells. *J Immunol.* (2006) 177:2195–207. 10.4049/jimmunol.177.4.2195 16887979

[25] Zhu X Schweitzer BL Romer EJ Sulentic CEW DeKoter RP. Transgenic expression of Spi-C impairs B-cell development and function by affecting genes associated with BCR signaling. *Eur J Immunol.* (2008) 38:2587–99. 10.1002/eji.200838323 18792411 PMC4457361

[26] Bednarski JJ Pandey R Schulte E White LS Chen BR Sandoval GJ RAG-mediated DNA double-strand breaks activate a cell type-specific checkpoint to inhibit pre-B cell receptor signals. *J Exp Med.* (2016) 213:209–23. 10.1084/jem.20151048 26834154 PMC4749927

[27] Soodgupta D White LS Yang W Johnston R Andrews JM Kohyama M RAG-mediated DNA breaks attenuate PU.1 activity in early B cells through activation of a SPIC-BCLAF1 complex. *Cell Rep.* (2019) 29:829.e–43.e. 10.1016/j.celrep.2019.09.026 31644907 PMC6870970

[28] Li SK Solomon LA Fulkerson PC DeKoter RP. Identification of a negative regulatory role for Spi-C in the murine B cell lineage. *J Immunol.* (2015) 194:3978–3807. 10.4049/jimmunol.1402432 25769919

[29] Oyake T Itoh K Motohashi H Hayashi N Hoshino H Nishizawa M Bach proteins belong to a novel family of BTB-basic leucine zipper transcription factors that interact with MafK and regulate transcription through the NF-E2 site. *Mol Cell Biol.* (1996) 16:6083–95. 10.1128/mcb.16.11.6083 8887638 PMC231611

[30] Watanabe-Matsui M Muto A Matsui T Itoh-Nakadai A Nakajima O Murayama K Heme regulates B-cell differentiation, antibody class switch, and heme oxygenase-1 expression in B cells as a ligand of Bach2. *Blood.* (2011) 117:5438–48. 10.1182/blood-2010-07-296483 21444915

[31] Igarashi K Itoh-Nakadai A. Orchestration of B lymphoid cells and their inner myeloid by Bach. *Curr Opin Immunol.* (2016) 39:136–42. 10.1016/j.coi.2016.01.012 26894991

[32] Muto A Tashiro S Nakajima O Hoshino H Takahashi S Sakoda E The transcriptional programme of antibody class switching involves the repressor Bach2. *Nature.* (2004) 429:566–71. 10.1038/nature02596 15152264

[33] Shinnakasu R Inoue T Kometani K Moriyama S Adachi Y Nakayama M Regulated selection of germinal-center cells into the memory B cell compartment. *Nat Immunol.* (2016) 17:861–9. 10.1038/ni.3460 27158841

[34] Muto A Ochiai K Kimura Y Itoh-Nakadai A Calame KL Ikebe D Bach2 represses plasma cell gene regulatory network in B cells to promote antibody class switch. *EMBO J.* (2010) 29:4048–61. 10.1038/emboj.2010.257 20953163 PMC3020649

[35] Kometani K Nakagawa R Shinnakasu R Kaji T Rybouchkin A Moriyama S Repression of the transcription factor Bach2 contributes to predisposition of IgG1 memory B cells toward plasma cell differentiation. *Immunity.* (2013) 39:136–47. 10.1016/j.immuni.2013.06.011 23850379

[36] Itoh-Nakadai A Hikota R Muto A Kometani K Watanabe-Matsui M Sato Y The transcription repressors Bach2 and Bach1 promote B cell development by repressing the myeloid program. *Nat Immunol.* (2014) 15:1171–80. 10.1038/ni.3024 25344725

[37] Itoh-Nakadai A Matsumoto M Kato H Sasaki J Uehara Y Sato Y A Bach2-cebp gene regulatory network for the commitment of multipotent hematopoietic progenitors. *Cell Rep.* (2017) 18:2401–14. 10.1016/j.celrep.2017.02.029 28273455

[38] Haldar M Kohyama M So AYL Kc W Wu X Briseño CG Heme-mediated SPI-C induction promotes monocyte differentiation into iron-recycling macrophages. *Cell.* (2014) 156:1223–34. 10.1016/j.cell.2014.01.069 24630724 PMC4010949

[39] Pfaffl M. Quantification strategies in real-time PCR Michael W. Pfaffl. In: Bustin SA editor. *A-Z Quant PCR.* La Jolla, CA: International University Line (2004). p. 87–112.

[40] Shi W Liao Y Willis SN Taubenheim N Inouye M Tarlinton DM Transcriptional profiling of mouse B cell terminal differentiation defines a signature for antibody-secreting plasma cells. *Nat Immunol.* (2015) 16:663–73. 10.1038/ni.3154 25894659

[41] Morita S Kojima T Kitamura T. Plat-E: an efficient and stable system for transient packaging of retroviruses. *Gene Ther.* (2000) 7:1063–6. 10.1038/sj.gt.3301206 10871756

[42] Afgan E Baker D van den Beek M Blankenberg D Bouvier D Cech M The Galaxy platform for accessible, reproducible and collaborative biomedical analyses: 2016 update. *Nucleic Acids Res.* (2016) 44:W3–10. 10.1093/nar/gkw343 27137889 PMC4987906

[43] Langmead B Trapnell C Pop M Salzberg SL. Ultrafast and memory-efficient alignment of short DNA sequences to the human genome. *Genome Biol.* (2009) 10:R25 10.1186/gb-2009-10-3-r25 PMC2690996 19261174

[44] Zhang Y Liu T Meyer CA Eeckhoute J Johnson DS Bernstein BE Model-based analysis of ChIP-Seq (MACS). *Genome Biol.* (2008) 9:R137 10.1186/gb-2008-9-9-r137 PMC2592715 18798982

[45] Shin H Liu T Manrai AK Liu XS. CEAS: cis-regulatory element annotation system. *Bioinformatics.* (2009) 25:2605–6. 10.1093/bioinformatics/btp479 19689956

[46] Huang da W Sherman BT. Lempicki RA. Systematic and integrative analysis of large gene lists using DAVID bioinformatics resources. *Nat Protoc.* (2009) 4:44–57. 10.1038/nprot.2008.211 19131956

[47] Ma W Noble WS Bailey TL. Motif-based analysis of large nucleotide data sets using MEME-ChIP. *Nat Protoc.* (2014) 9:1428–50. 10.1038/nprot.2014.083 24853928 PMC4175909

[48] Imanishi T Makela O. Strain differences in the fine specificity of mouse anti-hapten antibodies. *Eur J Immunol.* (1973) 3:323–30. 10.1002/eji.1830030602 4586168

[49] Carotta S Willis SN Hasbold J Inouye M Pang SHM Emslie D The transcription factors IRF8 and PU.1 negatively regulate plasma cell differentiation. *J Exp Med.* (2014) 211:2169–81. 10.1084/jem.20140425 25288399 PMC4203955

[50] Hasbold J Corcoran LM Tarlinton DM Tangye SG Hodgkin PD. Evidence from the generation of immunoglobulin G-secreting cells that stochastic mechanisms regulate lymphocyte differentiation. *Nat Immunol.* (2004) 5:55–63. 10.1038/ni1016 14647274

[51] Sciammas R Shaffer AL Schatz JH Zhao H Staudt LM Singh H. Graded Expression of Interferon Regulatory Factor-4 Coordinates Isotype Switching with Plasma Cell Differentiation. *Immunity.* (2006) 25:225–36. 10.1016/j.immuni.2006.07.009 16919487

[52] Miura Y Morooka M Sax N Roychoudhuri R Itoh-Nakadai A Brydun A Bach2 promotes B cell receptor-induced proliferation of B lymphocytes and represses cyclin-dependent kinase inhibitors. *J Immunol.* (2018) 200:2882–93. 10.4049/jimmunol.1601863 29540581

[53] Wang S Sun H Ma J Zang C Wang C Wang J Target analysis by integration of transcriptome and ChIP-seq data with BETA. *Nat Protoc.* (2013) 8:2502–15. 10.1038/nprot.2013.150 24263090 PMC4135175

[54] Yoshida H Lareau CA Ramirez RN Rose SA Maier B Wroblewska A The cis-regulatory atlas of the mouse immune system. *Cell.* (2019) 176:897.e–912.e. 10.1016/j.cell.2018.12.036 30686579 PMC6785993

[55] Oliver AM Martin F Kearney JF. Mouse CD38 is down-regulated on germinal center B cells and mature plasma cells. *J Immunol.* (1997) 158:1108–15. 9013949

[56] Dutra FF Bozza MT. Heme on innate immunity and inflammation. *Front Pharmacol.* (2014) 5:115 10.3389/fphar.2014.00115 PMC4035012 24904418

[57] Shinnakasu R Kurosaki T. Regulation of memory B and plasma cell differentiation. *Curr Opin Immunol.* (2017) 45:126–31. 10.1016/j.coi.2017.03.003 28359033

[58] Ochiai K Katoh Y Ikura T Hoshikawa Y Noda T Karasuyama H Plasmacytic transcription factor blimp-1 is repressed by Bach2 in B cells. *J Biol Chem.* (2006) 281:38226–34. 10.1074/jbc.M607592200 17046816

