# Supplementary material for: Opposing Roles for the Related ETS-Family Transcription Factors Spi-B and Spi-C in Regulating B Cell Differentiation and Function
Source: Front Immunol. 2020 May 8;11:841. doi: 10.3389/fimmu.2020.00841 (PMC7225353; doi:10.3389/fimmu.2020.00841)

Supplementary Table 1. Primer sequences used for qPCR analysis

Primer Name	Sequence (5’ – 3’)
Bach2 fwd	CAT CTC TTC CTC TGC CCA GT
Bach2 rev	AGA CAT GCC GTT CAA ACC AT
Bcl6 fwd	CCT GTG AAA TCT GTG GCA CTC G
Bcl6 rev	CGC AGT TGG CTT TTG TGA CG
Irf4 fwd	GAA AAT GGT TGC CAG GTG ACA G
Irf4 rev	GCT TCA GCA GAC CTT ATG CTT G
Pax5 fwd	TCG GAC CAT CAG GAC AGG ACA
Pax5 rev	GTT CCA CTA TCC TTT GGC GGA
Prdm1 fwd	ACA TAG TGA ACG ACC ACC CCT G
Prdm1 rev	CTT ACC ACG CCA ATA ACC TCT TTG
Tpb fwd	ACC GTG AAT CTT GGC TGT AAA C
Tbp rev	GCA GCA AAT CGC TTG GGA TTA
Spic fwd	AAA GGG AGG AAG AGG CAG GAG AAA
Spic rev	AAG TCT TTG GAG AAC AGC CTC GCT
ROI 1 cloning fwd	CCA CAT ATG TTA AAC ACC TCC TAT GT
ROI 1 cloning rev	ATT CAA ATC TCC TGA GCC AGT TAA T
ROI 1 site directed mutagenesis fwd	GAA AAA CAG GCC CTG TGC TTC GG
ROI 1 site directed mutagenesis rev	GGA AGA GAC TTG CAT TCA AAG
ROI 1 ChIP fwd	GTG GTC GAT AGG GCA GCT ATT T
ROI 1 ChIP rev	CAA CTC ACT CTC CGT GTT TCA
ROI ChIP 3 fwd	CGT AAG GCA GAG ATC CGA AGT
ROI ChIP rev	CTT CCT TAC TGA GAA CTC CCT GG
NCR ChIP fwd	ATA ACT ACT GTG GAT GCC CTC AC
NCR ChIP rev	CAG GCT TTA GAC GAT GCC TGA TA

Supplementary Figure 1. Antibody-secreting cell frequencies are similar between WT, *Spib*$^{-/-}$, and *Spib*$^{-/-}$*Spic*$^{+/-}$ for IgM, IgG2b, and IgG2c isotypes for both secondary and primary responses. (**A**) Frequencies of IgM-secreting ASCs per 10^6 splenocytes following immunization, boost at 30 days, and analysis at 37 days (data are shown for *n*=7 WT, and *n*=10 *Spib*$^{-/-}$ and *Spib*$^{-/-}$*Spic*$^{+/-}$ individual mice). (**B**) Frequencies of IgG2b-secreting ASCs per 10^6 splenocytes following immunization, boost at 30 days, and analysis at 37 days (data are shown for *n*=3 for WT, and *n*=5 *Spib*$^{-/-}$ and *Spib*$^{-/-}$*Spic*$^{+/-}$ individual mice). (**C**) Frequencies of IgG2c-secreting ASCs per 10^6 splenocytes following immunization, boost at 30 days, and analysis at 37 days (data are shown for *n*=3 WT, and *n*=5 *Spib*$^{-/-}$ and *Spib*$^{-/-}$*Spic*$^{+/-}$ individual mice). (**D**) Frequencies of IgM-secreting ASCs in naïve (right) and immunized (left) mice of the indicated genotypes following immunization and analysis at 7 days (data are shown for *n*=7 WT, *n*=8 *Spib*$^{-/-}$, and *n*=7 *Spib*$^{-/-}$*Spic*$^{+/-}$ individual mice). (**E**) Frequencies of IgG2b-secreting ASCs per 10^6 splenocytes following immunization and analysis at 7 days (data are shown for *n*=7 WT, and *n*=8 *Spib*$^{-/-}$ and *Spib*$^{-/-}$*Spic*$^{+/-}$ individual mice). (**F**) Frequencies of IgG2c-secreting ASCs per 10^6 splenocytes following immunization and analysis at 7 days (data are shown for *n*=7 WT, and *n*=8 for *Spib*$^{-/-}$ and *Spib*$^{-/-}$*Spic*$^{+/-}$ individual mice). Data are shown as mean ± SEM. Statistics were performed using one-way ANOVA (two-tailed). N.S. indicates not significant.

Supplementary Figure 1

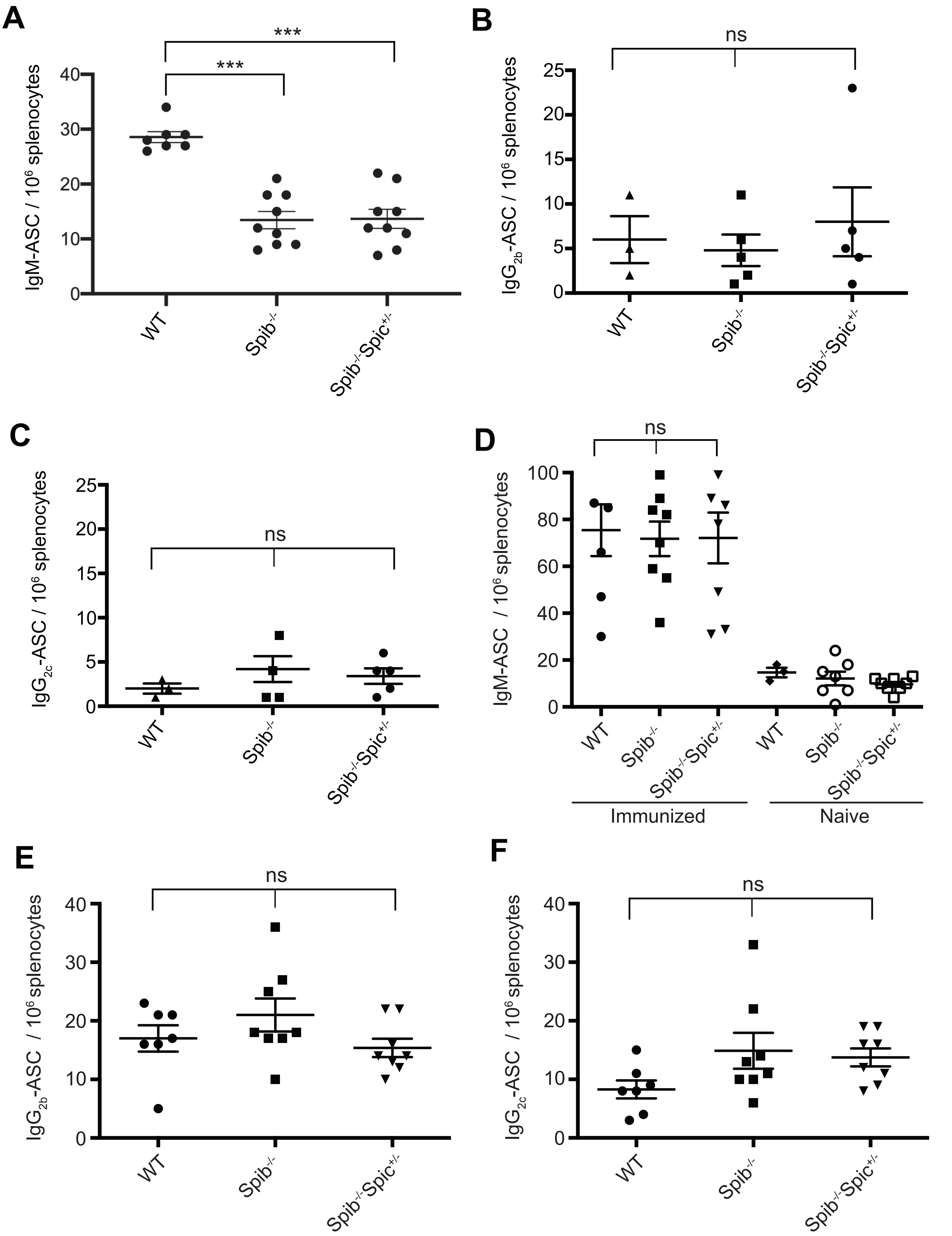

Supplement: Supplementary file 1 [file Data_Sheet_1.PDF]
